# Education and metabolic syndrome: a Mendelian randomization study

**DOI:** 10.3389/fnut.2024.1477537

**Published:** 2024-10-31

**Authors:** Dong Liu, Zi-Xiang Xu, Xue-lian Liu, Hai-Ling Yang, Ling-ling Wang, Yan Li

**Affiliations:** ^1^Emergency Department, China-Japan Union Hospital of Jilin University, Changchun, China; ^2^Pathology Department, Jilin Cancer Hospital, Changchun, China; ^3^Hematology Department, The First Hospital of Jilin University, Changchun, China; ^4^Stroke Center, Department of Neurology, The Affiliated Hospital of Beihua University, Changchun, China

**Keywords:** education, metabolic syndrome, waist circumference, hypertension, fasting blood glucose, triglycerides, high-density lipoprotein cholesterol

## Abstract

**Aims:**

The metabolic syndrome (MetS), a collection of conditions that heighten the risk of disease development and impose economic burdens on patients. However, the causal relationship between education and MetS was uncertain. In this study, the Mendelian randomization (MR) method was employed to elucidate the potential causal link between education and the MetS and its components.

**Method:**

Single nucleotide polymorphisms (SNPs) associated with education, MetS, and its components were sourced from a public database, with the inverse variance-weighted (IVW) method utilized for analysis.

**Results:**

Education demonstrated a significant negative correlation with the risk of MetS (OR = 0.55, 95% CI = 0.48–0.63, *p* = 2.18E−51), waist circumference(OR = 0.80, 95% CI = 0.76–0.83, *p* = 4.98E-33), hypertension (OR = 0.96, 95% CI = 0.95–0.97; *p* = 4.54E-10), Fasting blood glucose (OR = 0.94, 95% CI = 0.91–0.97, *p* = 7.58E-6) and triglycerides (OR = 0.83, 95% CI = 0.79–0.87, *p* = 7.87E-18) while showing a positive association with high-density lipoprotein cholesterol (OR = 1.22, 95% CI = 1.18–1.25, *p* = 1.45E-31).

**Conclusion:**

The findings of this study suggest that education can decrease the incidence of MetS.

## 1 Introduction

In the global context, the prevalence of metabolic syndrome (MetS) had ranged from 20 to 25% and exhibited gradually increased over the past several years (1, 2). MetS had been associated with an elevated risk of developing various illnesses, including a 29% increase in the risk of major coronary events, a 30% increase in the risk of recurrent myocardial infarction, and a 12% increase in the risk of dementia (3, 4). Meanwhile, MetS had been linked to a rising mortality rate, with a 24% increase in all-cause mortality and a 44% increase in mortality due to heart disease (5).

The level of education, as an indicator of socioeconomic status, has the potential to impact both the prevalence of cardiovascular disease and the prevalence of MetS (6, 7). Individuals with a college education demonstrated a 57% lower risk of developing MetS than those with only a primary school education (8). Recent research findings had indicated that while several studies had explored the association between education and MetS, a definitive causal relationship between the two had not been found.

Mendelian randomization (MR) was initially introduced by Katan in 1986, utilizing genome-wide association study(GWAS) data to employ single nucleotide polymorphisms(SNPs) as instrumental variables(IVs) for investigating causal relationships between exposure and outcome (9). This method distinguishes itself from others by its independence from confounding variables and ability to avoid reverse causation (10).

This study utilized a 2-sample MR method to estimate the association between education and MetS.

## 2 Method

### 2.1 Overview of the study design

As the assessment criteria outlined by the International Diabetes Federation (IDF) and the American Heart Association/National Heart, Lung, and Blood Institute (AHA/NHLBI) (11), five components (waist circumference (WC), hypertension, fasting blood glucose(FBG), triglycerides(TG), and high-density lipoprotein cholesterol(HDL-C)) were identified as elements of MetS.

**Figure 1:**
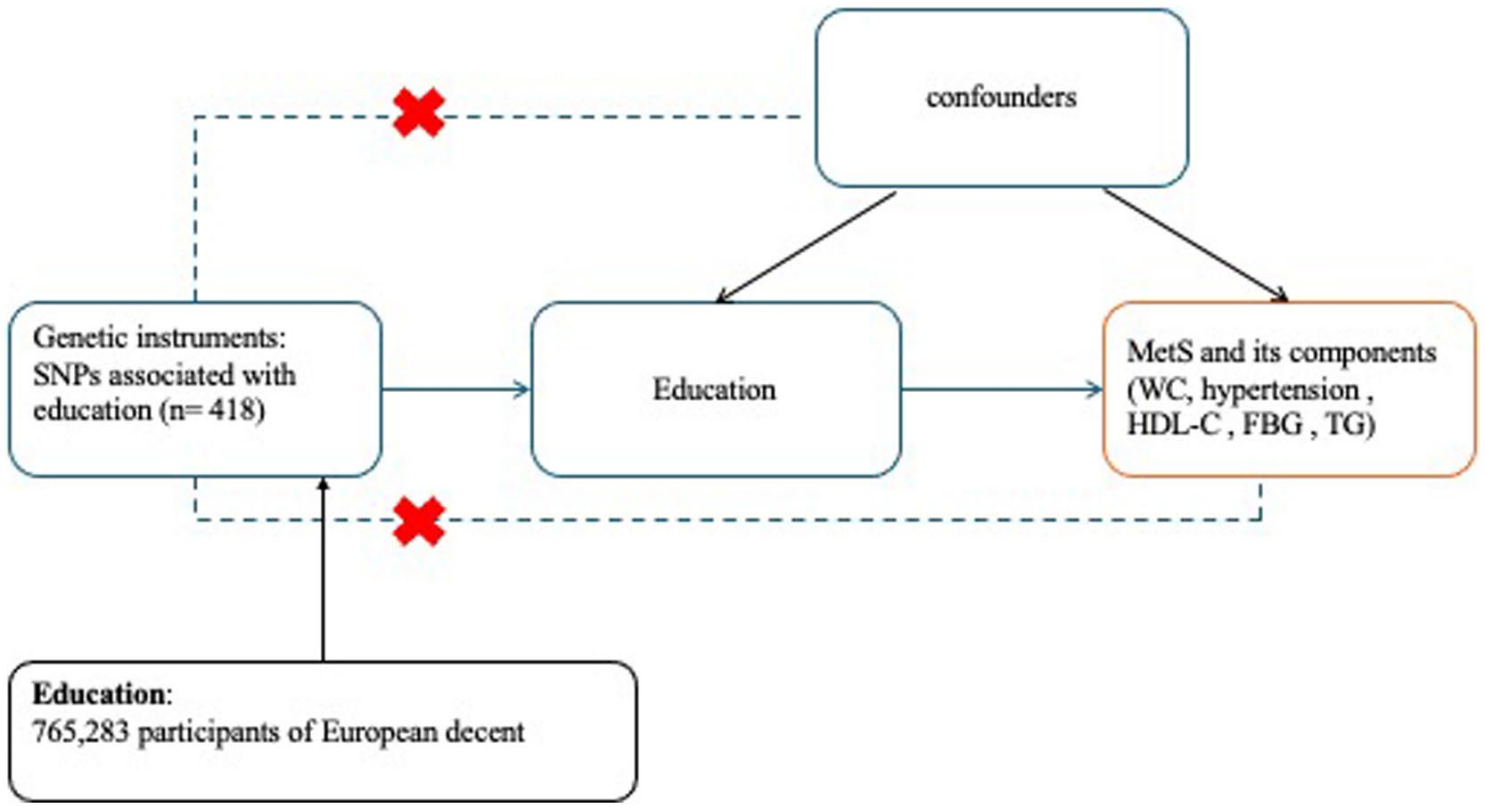
Overview of the study design in this MR study. MR, Mendelian randomization; SNP, single-nucleotide polymorphism; MetS, metabolic syndrome, FBG, fasting blood glucose, TG, triglycerides, WC, waist circumference, HDL-C, high-density lipoprotein cholesterol.

In this research, a 2-sample MR analysis was conducted. The initial phase involved investigating the impact of education on MetS and WC, hypertension, FBG, TG, and HDL-C. The selection of IVs shown in Figure 1 was guided by three key assumptions: (1) the genetic instruments are strongly correlated with the exposure variable; (2) the genetic instruments are not affected by any confounders; and (3) the genetic instruments solely influence the outcome through the exposure variable. The study design is depicted in Figure 1.

### 2.2 Selection of genetic instruments for MR analyses

**Figure 2:**
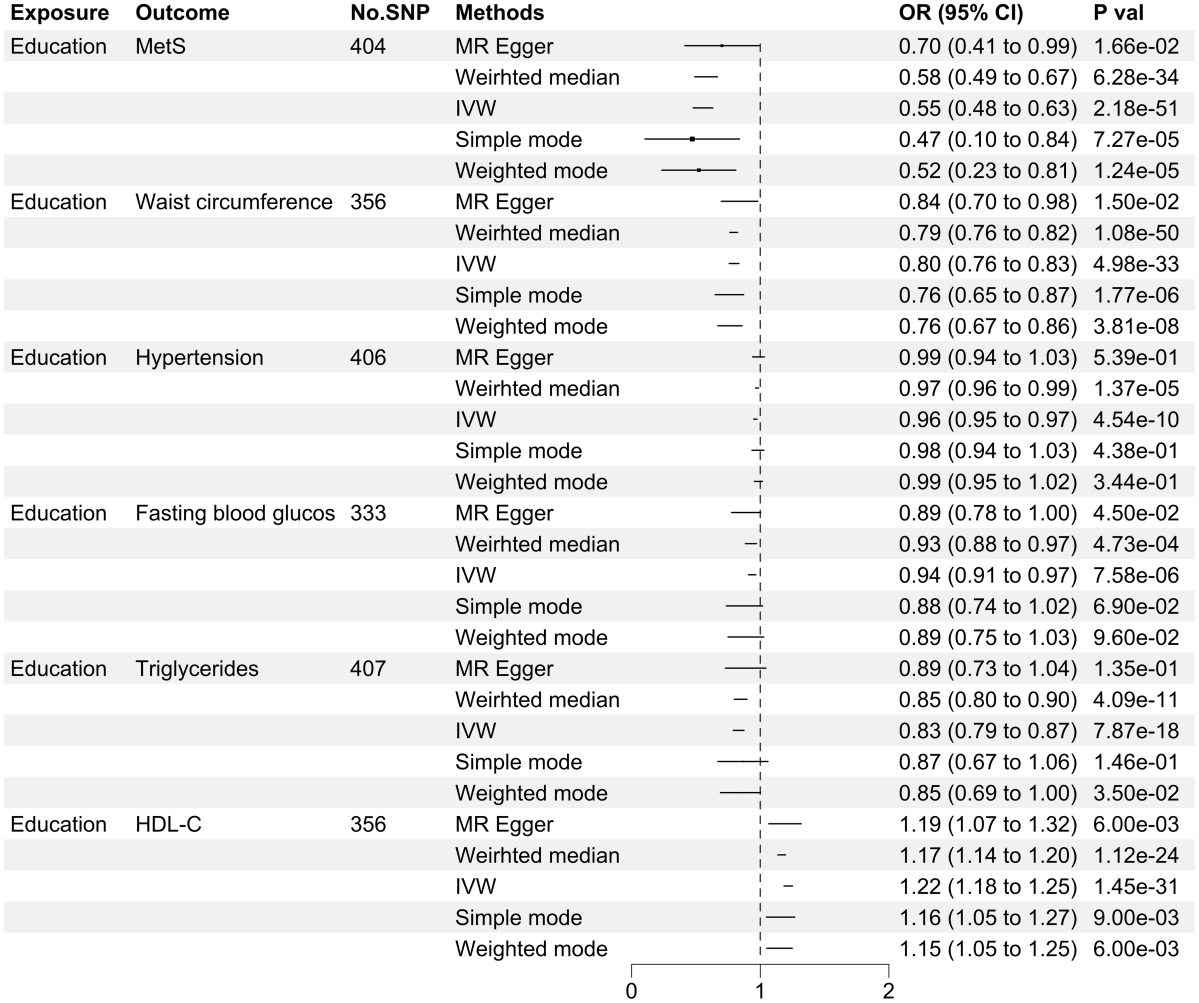
Genetic predicated MetS and its components on the risk of education in the MR analysis.

The criteria for selecting IVs in this study included the following: (1): only SNPs with *p* values less than 5 × 10–8 were included in the study; (2); SNPs with linkage disequilibrium (LD) based on a specified *R*^2^ threshold of 0.001 and a clumping distance of 10,000 kb were removed; and (3) the F statistic was calculated to assess the predictive potential of the IVs, with a threshold of F statistic >10 indicating strong predictive ability (Figure 2).

### 2.3 Data sources for education

The largest published GWAS for education was obtained from the Social Science Genetic Association Consortium (SSGAC), encompassing a total of 765,283 individuals of European ancestry (12). A total of 418 SNPs associated with WC with *p* values less than 5 × 10–8 were included in the study.

### 2.4 Data sources for MetS and its components

The genetic variation data for MetS were acquired from the GWAS, which includes a total of 291,107 individuals of European descent (comprising 59,677 cases and 231,430 controls) (13).

A summary of GWASs for WC was obtained from the Medical Research Council (MRC) Integrative Epidemiology Unit (IEU) GWAS pipeline, which uses a sample size of 407,661 individuals (14).

For hypertension, genetic data were extracted from the MRC-IEU UK Biobank GWAS pipeline, and a total of 484,598 subjects of European ancestry (129,909 cases and 354,689 controls) were included in this study (15).

The summary statistics for FBG, also taken from the MRC-IEU UK Biobank pipeline, included 58,074 individuals of European ancestry (16).

For TG, the IVs were obtained from the MRC-IEU UK Biobank pipeline, which included data from a cohort of over 115,082 individuals (17).

Similar to HDL-C, summary-level statistics were extracted from the MRC-IEU UK Biobank pipeline, which included data from a cohort of over 357,810 subjects of white British descent (14).

### 2.5 MR analysis method

Inverse variance-weighted (IVW), MR–Egger, weighted median, simple mode, and weighted mode were employed in 2-sample MR analysis to assess the association between education and MetS. The IVW method was applied in the primary analysis, involving the combination of Wald ratios of individual SNPs to derive an overall causal estimate. MR–Egger, weighted median, simple mode, and weighted mode methods were utilized for alternative analyses. MR egger was used for directional horizontal pleiotropy. MR–Egger, IVW methods, Cochran’s Q statistics and funnel plots were employed for the assessment of heterogeneity (10). Sensitivity analysis was performed through leave-one-out (LOO) analysis. TwoSample MR packages in R (version 4.3.3) were used to perform all the statistical analyses. The web tool^1^ was utilized to calculate the power for MR. *p* values <0.004 (0.05/6 = 0.008), as determined by Bonferroni correction, were used in this analysis. Significance was defined as *p* < 0.05 in the MR–Egger test and heterogeneity test.

## 3 Results

Among the 418 education-associated variants, 3 SNPs were not available for MetS; one SNP was not available for WC, hypertension or HDL-C; and 76 SNPs were not available for FBG, which is a no missing variant in triglycerides. Palindromic SNPs were excluded from the analysis. A total of 404 SNPs were utilized as genetic instruments for MetS, 356 for WC, 406 for hypertension, 333 for FBG, 407 for TG and 356 for HDL-C in this study. The *R*^2^ and F statistics were calculated to assess the efficacy of the genetic instruments (Table 1).

**Table 1:** *R*^2^ and F- statistics for genetic instruments and the power for MR.

Exposure	Outcome	No.SNP	*R* ^2^	F-statistic	Power
Education	Metabolic syndrome	404	0.02837132	53.81669	100
Education	Waist circumference	356	0.02845168	53.71459	100
Education	hypertension	406	0.02845168	53.71459	56.9
Education	Fasting blood glucos	333	0.02376203	54.44138	100
Education	Triglycerides	407	0.02849483	53.66967	100
Education	High density lipoprotein cholesterol levels	356	0.02845168	53.71459	100

The results were shown in Table 2 and Figure 1. The scatter plots were presented in Supplementary Figure S3. The forest plots were presented in Supplementary Figure S4. The results of the IVW analysis [odds ratio (OR) = 0.55, 95% confidence interval (CI) = 0.48–0.63, *p* = 2.18E−51], MR–Egger regression (OR = 0.70, 95% CI = 0.41–0.99, *p* = 1.66E−2) and weighted median analysis (OR = 0.58, 95% CI = 0.49–0.67, *p* = 6.28E−34) indicated each one-s.d.-higher education level was negatively association with MetS (Table 2). The IVW analysis yielded Cochran’s Q test *p* values (*p* < 0.001), suggesting substantial heterogeneity in the results. However, the funnel plot did not display asymmetry (Supplementary Figure S1), indicating no evidence of publication bias. Furthermore, MR–Egger intercept values (intercept = −0.003; *p* = 0.098) indicate the absence of significant horizontal pleiotropy in this study. Furthermore, the LOO analysis demonstrated no significant changes upon the removal of any IVs (Supplementary Figure S2).

**Table 2:** Geneticly predicted effect of education on the risk of MetS and its components in the MR analysis.

Exposure	Outcome	No.SNP	Methods	OR(95%CI)	*p*- val	Egger_intercept	p-Egger_intercept
Education	MetS	404	MR Egger	0.70 (0.41,0.99)	1.66 × 10^−2^	−0.003	0.098
			Weighted median	0.58 (0.49,0.67)	6.28 × 10^−34^		
			IVW (Q = 918.24, *p* < 0.001)	0.55 (0.48,0.63)	2.18 × 10^−51^		
			Simple mode	0.47 (0.10,0.84)	7.27 × 10^−05^		
			Weighted mode	0.52 (0.23,0.81)	1.24 × 10^−05^		
Education	Waist circumference	356	MR Egger	0.84 (0.70,0.98)	0.015	−0.001	0.456
			Weighted median	0.79 (0.76,0.82)	1.08 × 10^−50^		
			IVW(Q = 2088.63, *p* < 0.001)	0.80 (0.76,0.83)	4.98 × 10^−33^		
			Simple mode	0.76 (0.65,0.87)	1.77 × 10^−06^		
			Weighted mode	0.76 (0.67,0.86)	3.81 × 10^−08^		
Education	Hypertension	406	MR Egger	0.99 (0.94,1.03)	0.539	−0.000	0.284
			Weighted median	0.97 (0.96,0.99)	1.37 × 10^−05^		
			IVW(Q = 1434.8, *p* < 0.001)	0.96 (0.95,0.97)	4.54 × 10^−10^		
			Simple mode	0.98 (0.94,1.03)	0.438		
			Weighted mode	0.99 (0.95,1.02)	0.344		
Education	Fasting blood glucos	333	MR Egger	0.89 (0.78,1.00)	0.045	0.001	0.361
			Weighted median	0.93 (0.88,0.97)	4.73 × 10^−4^		
			IVW(Q = 363.74, *p* = 0.111)	0.94 (0.91,0.97)	7.58 × 10^−6^		
			Simple mode	0.88 (0.74,1.02)	0.069		
			Weighted mode	0.89 (0.75,1.03)	0.096		
Education	Triglycerides	407	MR Egger	0.89 (0.73,1.04)	0.135	−0.001	0.413
			Weighted median	0.85 (0.80,0.90)	4.09 × 10^−11^		
			IVW(Q = 751.71, *p* < 0.001)	0.83 (0.79,0.87)	7.87 × 10^−18^		
			Simple mode	0.87 (0.67,1.06)	0.146		
			Weighted mode	0.85 (0.69,1.00)	0.035		
Education	HDL-C	356	MR Egger	1.19 (1.07,1.32)	0.006	0.000	0.730
			Weighted median	1.17 (1.14,1.20)	1.12 × 10^−24^		
			IVW(Q = 1473.60, *p* < 0.001)	1.22 (1.18,1.25)	1.45 × 10^−31^		
			Simple mode	1.16 (1.05,1.27)	0.009		
			Weighted mode	1.15 (1.05,1.25)	0.006		

In terms of its constituent elements, each one-s.d.-higher education level was negatively correlated with WC, hypertension, FBG, and triglycerides but positively correlated with HDL-C. In the IVW model, the ORs with 95% CIs per log-odds increase in genetically predicted education level were 0.80 (95% CI: 0.76–0.83; *p* = 4.98E–33) for WC, 0.96 (95% CI: 0.95–0.97; *p* = 4.54E–10) for hypertension, 0.94 (95% CI: 0.91–0.97; *p* = 7.58E–6) for FBG, 0.83 (95% CI: 0.79–0.87; *p* = 7.87E–18) for triglycerides, and 1.22 (95% CI, 1.18–1.25; *p* = 1. 45E–31) for HDL-C (see Table 2). The findings were consistent across the various models. Potential horizontal pleiotropy was not found in the IVW model (all *p* values >0.05), as shown in Table 2. The results of the Cochran Q test revealed significant heterogeneity in WC, hypertension, TG, and HDL-C, but not FBG. Additionally, analysis of the funnel plot (Supplementary Figure S1) did not detect any visible asymmetry, suggesting a lack of evidence for heterogeneity. Furthermore, the stability of the results remained consistent even after the exclusion of any single SNP through LOO analysis. (Supplementary Figure S2).

## 4 Discussion

This is the first article to elucidate the causal relationship between education and metabolic syndrome, using Mendelian randomization method. In this 2-sample MR study, each addition 4.2 years of education decreased 45% risk of MetS, had a negative association with WC, hypertension, FBG, and TG, a positive association with HDL-C.

Higher education levels are linked to improved health habits and lower rates of MetS, as well as a decreased risk of cardiovascular and cerebrovascular diseases such as heart disease and stroke (18–20). A cross-sectional analysis revealed a negative association between education and MetS (21), which is consistent with the findings of our own study. A study conducted in Korea demonstrated that education level has both direct and indirect impacts on MetS, with higher levels of education indirectly influencing food choices to decrease the prevalence of MetS. Specifically, individuals with higher education levels were more likely to adopt healthier food habits, such as consuming fruits, vegetables, and milk, with this effect being more pronounced in females (21). Previous research has revealed sex differences in the onset and progression of MetS. Interestingly, these differences also extend to the association between education level and MetS, with a more pronounced effect observed in females (8). A study focusing on a cohort of middle-aged women revealed that higher education levels were associated with a decreased prevalence of MetS. Specifically, individuals who had completed college or higher were 2.7 times more likely to have MetS (22). Prior research has generally posited that education is among the factors influencing metabolic syndrome. However, our study indicates that higher levels of education are associated with a reduced incidence of metabolic syndrome.

As the most important component of MetS, WC can also be influenced by education level. In the European cohort, higher levels of education were found to have a significant effect on reducing WC (23). A cross-sectional study conducted on elderly adults in Taipei further supports this finding, showing a positive correlation between obesity and lower education levels, particularly among females, with no significant association with income level (24). These results are consistent with our own study, which revealed a negative relationship between years of education and WC. In a prior study conducted by Jose P. Lopez-Lopez et al., the prevalence of hypertension was significantly higher among populations of African origin than among other populations. Additionally, the study identified an association between the risk of hypertension and lower levels of education (25). Another MR study also identified a lower level of education as a risk factor for hypertension (26). This finding is consistent with the results of our study. A study conducted by Braverman-Bronstein et al. demonstrated that the association between education level and diabetes is modulated by sex and region (27). Specifically, a negative correlation was detected in females, whereas males presented varying correlations across different regions (27). The study revealed a negative correlation in countries such as Argentina, Brazil, Colombia, Chile, and Mexico, whereas a positive correlation was observed in Peru, Panama, and El Salvador (27). A propensity score-based analysis conducted in Bangladesh revealed no significant influence of education level on the prevalence of diabetes and hypertension (28). This finding conflicts with our study. In our study, we observed that a longer education duration was associated with a lower risk of diabetes and hypertension. The discrepancies in findings across studies may be attributed to variations in study populations. A study conducted by Christopher R. Stephens demonstrated that an increase in educational duration was associated with a reduction in WC, blood pressure, FBG, and TG, alongside an increase in HDL-C. However, subgroup analysis revealed that WC increased with education duration exclusively in females (20). Additionally, a cross-sectional analysis of an Australian cohort by L. A. Simons indicated that TG and HDL-C levels were negatively correlated with educational attainment in females but not in males (29). In our study, we observed that extended educational duration was associated with increased WC, FBG, TG, and hypertension risk, as well as decreased HDL-C levels.

## 5 Strengths

First, MR was used in this study, reducing the influence of confounders. Second, GWAS data include comprehensive and summary-level data for exposure (education) and outcome (MetS, WC, hypertension, FBG, TG, HDL-C) and therefore have high investigative power. Second, multiple statistical methods were used in this study, increasing the credibility of the results.

## 6 Limitations

First, the influence of IVs on outcomes remains uncertain. Second, owing to the unavailability of data (For example, GWAS data for education is available only for overall population, not separated by sex), subgroup analyses, such as examinations of sex differences, could not be conducted. Finally, significant heterogeneity was identified in this study on the basis of the Cochran’s Q value, but LOO analysis revealed that the result was credible.

## 7 Conclusion

This study employed MR methodology to investigate the association between levels of education and MetS. A low-level education population should be focused on reducing the incidence of MetS, obesity, hypertension, FBG, TG and HDL-C.

## Data Availability

Publicly available datasets were analyzed in this study. This data can be found at: https://thessgac.com/papers/3; https://gwas.mrcieu.ac.uk/datasets/?trait__icontains=ebi-a-GCST90014020; https://gwas.mrcieu.ac.uk/datasets/ebi-a-GCST90038604/; https://gwas.mrcieu.ac.uk/datasets/ebi-a-GCST005186/; https://gwas.mrcieu.ac.uk/datasets/ebi-a-GCST90092992/; https://gwas.mrcieu.ac.uk/datasets/ebi-a-GCST90014007/; https://www.liebertpub.com/doi/10.1089/met.2019.0070.

## Footnotes

^1^ https://sb452.shinyapps.io/power/
