# Supplementary material for: Education and metabolic syndrome: a Mendelian randomization study
Source: Front Nutr. 2024 Oct 31;11:1477537. doi: 10.3389/fnut.2024.1477537 (PMC11562850; doi:10.3389/fnut.2024.1477537)
Supplement: Supplementary file 1 [file Image_1.pdf]

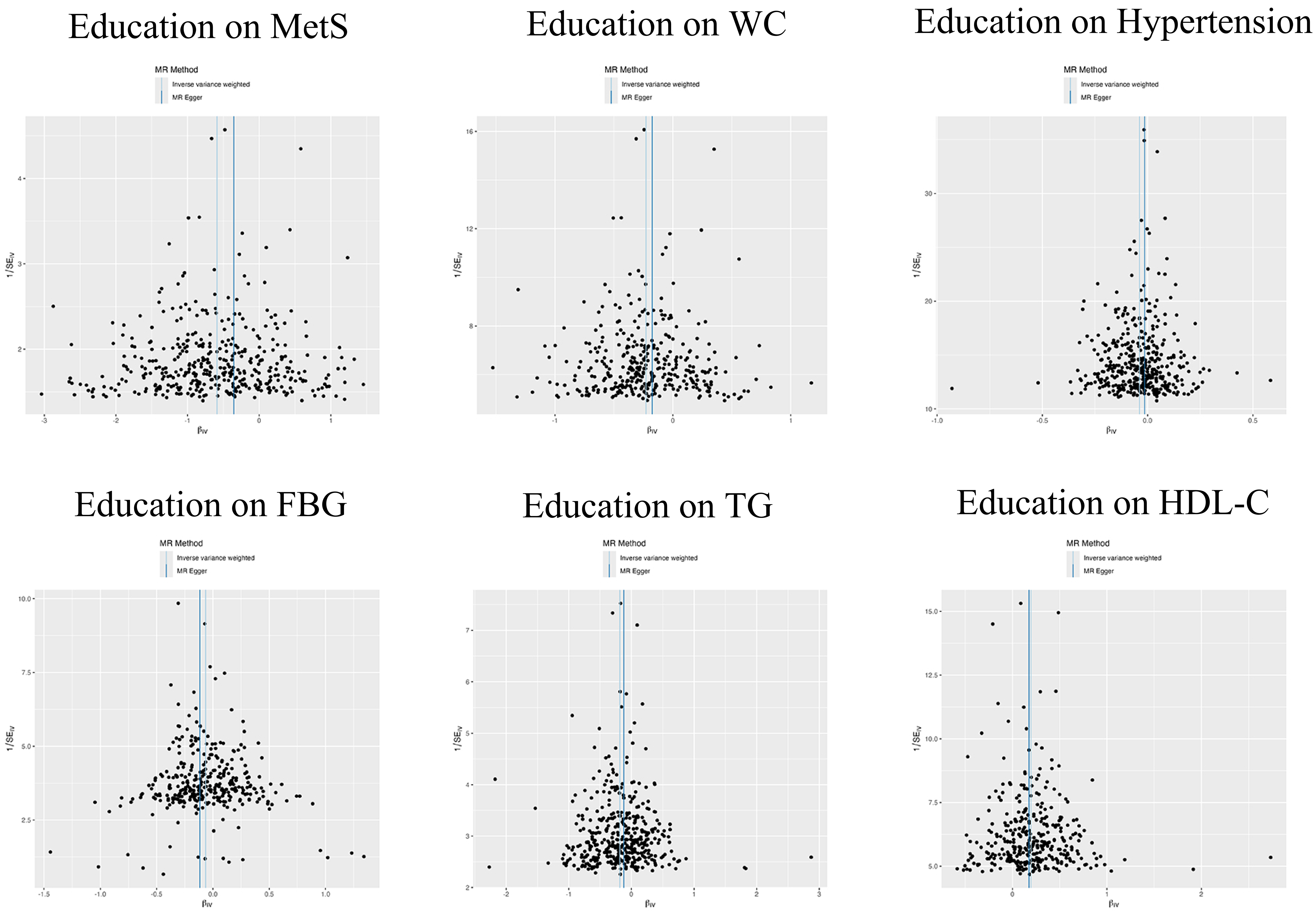

Supplementary Fig1. The funnel plot of the association between genetically predicted education on MetS and its components in MR analysis. MetS metabolic syndrome, FBG fasting blood glucose, TG triglycerides, WC waist circumference, HDL-C high-density lipoprotein cholesterol.
