# Supplementary material for: Education and metabolic syndrome: a Mendelian randomization study
Source: Front Nutr. 2024 Oct 31;11:1477537. doi: 10.3389/fnut.2024.1477537 (PMC11562850; doi:10.3389/fnut.2024.1477537)
Supplement: Supplementary file 3 [file Image_3.pdf]

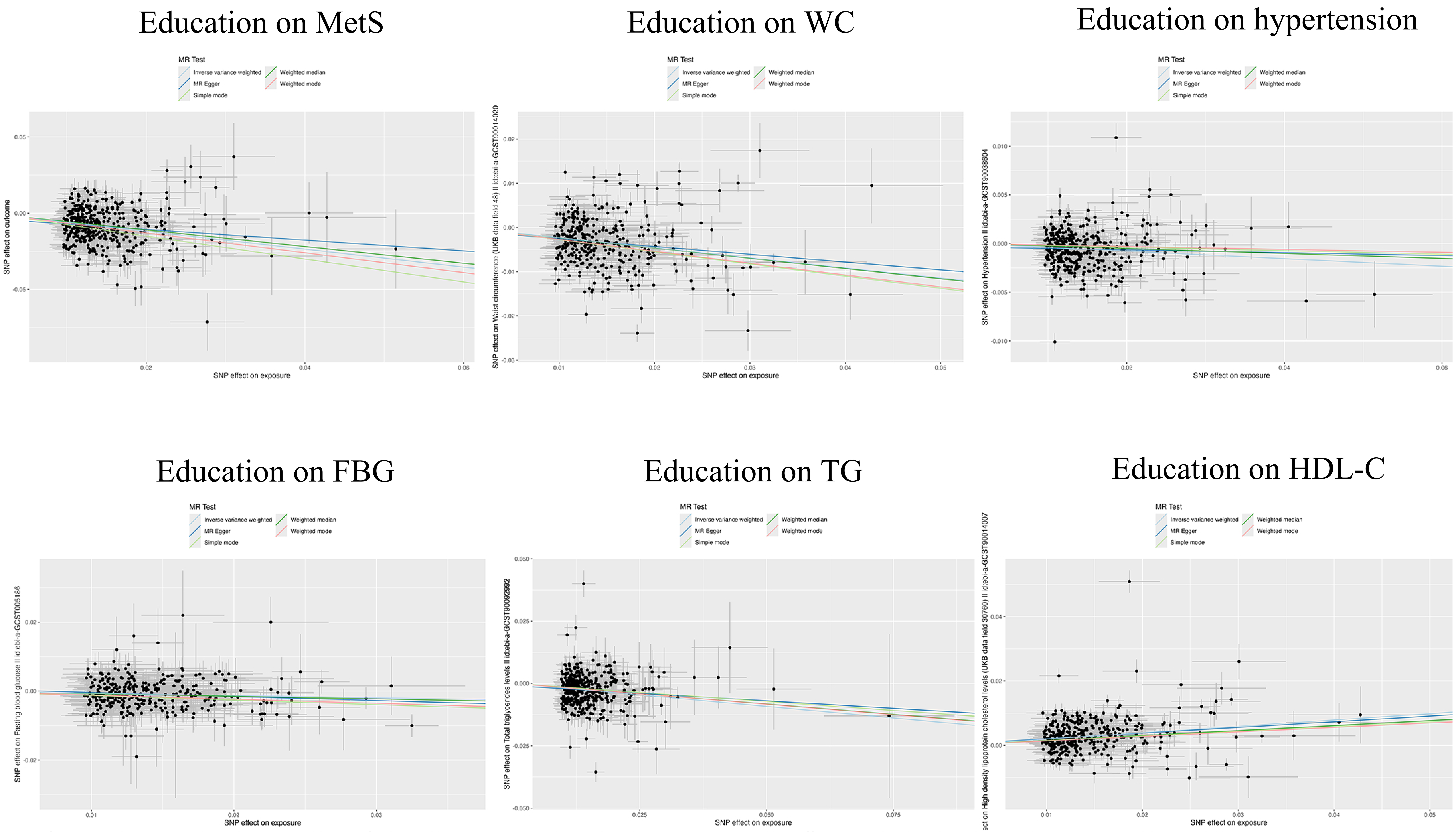

Supplementary Fig3. The scatter plot of the association between genetically predicted education on MetS and its components
